# Risk prediction model of frailty and its associated factors in older adults: a cross-sectional study in Anhui Province, China

**DOI:** 10.3389/fnut.2025.1611914

**Published:** 2025-07-17

**Authors:** Xu Qin, Huan Liu, Xiubin Tao, Zhiqing Zhou, Guangliang Mei, Ming Zhang, Shengqiang Zou

**Affiliations:** ^1^School of Medicine, Jiangsu University, Zhenjiang, Jiangsu, China; ^2^Department of the Interventional, The First Affiliated Hospital of Wannan Medical College (Yijishan Hospital of Wannan Medical College), Wuhu, Anhui, China; ^3^Department of Hemodialysis, The First Affiliated Hospital of Wannan Medical College (Yijishan Hospital of Wannan Medical College), Wuhu, Anhui, China; ^4^Department of Nursing, The First Affiliated Hospital of Wannan Medical College (Yijishan Hospital of Wannan Medical College), Wuhu, Anhui, China; ^5^The Department of Party Affairs, The First Affiliated Hospital of Wannan Medical College (Yijishan Hospital of Wannan Medical College), Wuhu, Anhui, China; ^6^School of Innovation and Entrepreneurship, Wanna Medical College, Wuhu, Anhui, China; ^7^School of Educational Science, Anhui Normal University, Wuhu, Anhui, China; ^8^Department of Critical Care Medicine, The Third People's Hospital of Zhenjiang (The Third Affiliated Hospital of Jiangsu University), Zhenjiang, Jiangsu, China

**Keywords:** frailty, older adults, prediction model, Anhui Province (China), cross-sectional study

## Abstract

**Background:**

In the context of aging in China, frailty has become a major public health challenge, placing an enormous economic burden on both society and families. Frailty can trigger serious adverse effects on the physical and mental health of older adults. It highlights the urgent requirement for addressing the issue of frailty among older adults. Accordingly, the present study was conducted to identify potential risk factors and develop a validated risk predictive model for frailty in older Chinese adults.

**Methods:**

Following a cross-sectional design, the present study selected participants from Anhui Province, China, using convenience sampling. Eligible data were collected using a demographic questionnaire, the Fatigue, Resistance, Ambulation, Illnesses, & Loss of Weight (FRAIL) scale, the strength, assistance walking, rise from a chair, climb stairs, and falls (SARC-F) scale, the social FRAIL scale, and the short-form mini-nutritional assessment (MNA-SF). Furthermore, a one-way analysis of variance and a multivariate analysis were utilized to identify the optimal predictive factors of the model. The logistic regression model was used to explore frailty-associated factors in older Chinese adults. Finally, a nomogram was constructed to establish the predictive model, with the application of calibration curves to evaluate the accuracy of the nomogram. The area under the receiver operating characteristic (ROC) curve (AUC) and decision curve analysis (DCA) were used to evaluate the performance of prediction.

**Results:**

Our final analysis incorporated 1,611 older Chinese adults who completed the questionnaire, with the incidence of frailty found in 491 (30.5%) cases. Multivariate logistic regression analysis showed that age, sarcopenia, malnutrition, social frailty, and hospitalization within the past 6 months were predictors of frailty. Consequently, the resultant nomogram demonstrated good consistency and accuracy. The AUC values of the model and the internal validation set were 0.86 (95%CI: 0.84–0.89) and 0.89 (95%CI: 0.85–0.92), respectively (both *p* > 0.05 via the Hosmer–Lemeshow test). In addition, the calibration curve showed significant agreement between the nomogram predictions and the observed values. ROC and DCA analyses revealed good predictive performance of the nomogram.

**Conclusion:**

This study constructs a frailty risk predictive model with good consistency and predictive performance, facilitating an effective prediction of the onset of frailty among older Chinese adults. It may benefit the screening of high-risk populations and the implementation of early interventions clinically.

## Introduction

China has recently become the country with the largest number of older adults globally, with a remarkable increase in the aging population. According to the seventh national population census of the People’s Republic of China, there were 260,020,000 ≥ 60-year-old older adults as of 2020, representing 18.7% of the total population (1). This percentage represents a 5.44% increase in the proportion of individuals aged ≥60 compared to 2010, posing major public health challenges, particularly regarding frailty in older adults (2). This trend may have a remarkable negative influence on the physical health of older adults and may significantly impact clinical practice and public health systems (3).

Frailty refers to a complex geriatric syndrome in certain individuals with decreased physiological reserves, resulting in increased vulnerability and susceptibility to stressors. Frail older adults have been reported to experience a higher risk of falls, disabilities, hospitalization, and mortality, placing a heavy burden on the medical and health system (4, 5). With multifactorial etiologies, frailty is primarily attributed to physiological, psychological, and social factors (2). It highlights the necessity of investigating the prevalence of frailty and its associated influential factors among older Chinese adults to guide public health strategies and formulate public health policies. It can further boost the identification of potentially modifiable risk factors and support the early implementation of targeted primary prevention measures, thereby mitigating or reversing the prevalence of frailty in older adults.

Frailty, featured as a dynamic process, can precede the onset of disability and can be prevented, delayed, or even reversed through proactive interventions (6). Given its complex pathogenesis, a holistic approach that considers personal, biological, interpersonal, and psychological factors may be of great significance for conducting a predictive modeling study on the influences of frailty in older adults (7).

Among the relevant factors, malnutrition has been recognized as a predominant risk factor for frailty in older adults. Leij-Halfwerk S et al. (8) reported that approximately one-quarter of older adults (≥65 years old) were malnourished or at risk of malnutrition. Other scholars have also supported the association between malnutrition and the occurrence or increased possibility of frailty (9). Therefore, to reduce frailty among older adults through nutritional intervention programs, it is important to understand the correlation between nutritional status and frailty.

Furthermore, similar to frailty, sarcopenia has become a major geriatric concern in the context of aging in China. Landi et al. even suggested that sarcopenia could serve as a biomarker of frailty. Wang C et al. pointed out that “sarcopenia” is an independent risk factor for frailty in older adults with chronic kidney disease (10). Current research on sarcopenia and frailty in older adults has been dominated by assessments using the Asian Working Group for Sarcopenia 2019 (AWGS 2019) criteria. However, to date, limited data exist on the use of the SARC-F scale to screen older adults for sarcopenia and to further explore the relationship between sarcopenia and frailty progression.

Generally, social frailty is a useful concept that reveals a series of social functions. According to its general definition, it is a state of being at risk of losing, or having lost, social resources, social behaviors, or activities, all of which are needed to meet an individual’s basic social needs (11). More importantly, social frailty is strongly associated with many adverse health outcomes, such as disability, mortality, and motoric cognitive risk syndrome (12–14). It is well known that social factors are irreplaceable in maintaining physical, cognitive, and psychological functioning, as well as the ability to perform daily activities in older adults (15). Moreover, older adults may experience a significantly higher incidence of social frailty. Despite the existence of numerous cross-sectional studies on social frailty among older adults, there is currently a lack of research on the predictive modeling of social frailty and frailty in this population. In particular, in China, limited information is available on the relationship between social frailty and frailty (16), especially due to the lack of large-sample, multicenter cross-sectional studies. As a result, to effectively address this gap in the literature, a large-scale cross-sectional study is needed to better decipher social frailty and frailty in older adults.

Grounded in existing evidence, the present study proposed a hypothesis that malnutrition, sarcopenia, social frailty, and hospitalization within the past 6 months may be associated with frailty in older adults. For further verification and expanded investigation, this study was conducted to identify key factors associated with frailty in older adults, with the purpose of providing valuable insights into the prevention and intervention of frailty in this population. It is anticipated that our study will offer valuable references for reducing the prevalence of frailty in older adults and improving their overall health.

## Materials and methods

### Study design

In strict accordance with the Strengthening the Reporting of Observational Studies in Epidemiology (STROBE) reporting guidelines, we conducted a large-scale descriptive cross-sectional study. Eligible older adults were recruited in Anhui Province, eastern China, using convenience sampling from December 2023 to May 2024. Before the formal investigation, all investigators involved in this study underwent unified, standardized training to clarify the scoring criteria of the survey scale, communication skills, and precautions. Researchers were responsible for distributing self-made electronic questionnaires to each participant after obtaining their informed consent. Moreover, they conducted face-to-face interviews and assisted participants with lower educational levels or reading difficulties in completing the questionnaires.

**Figure 1:**
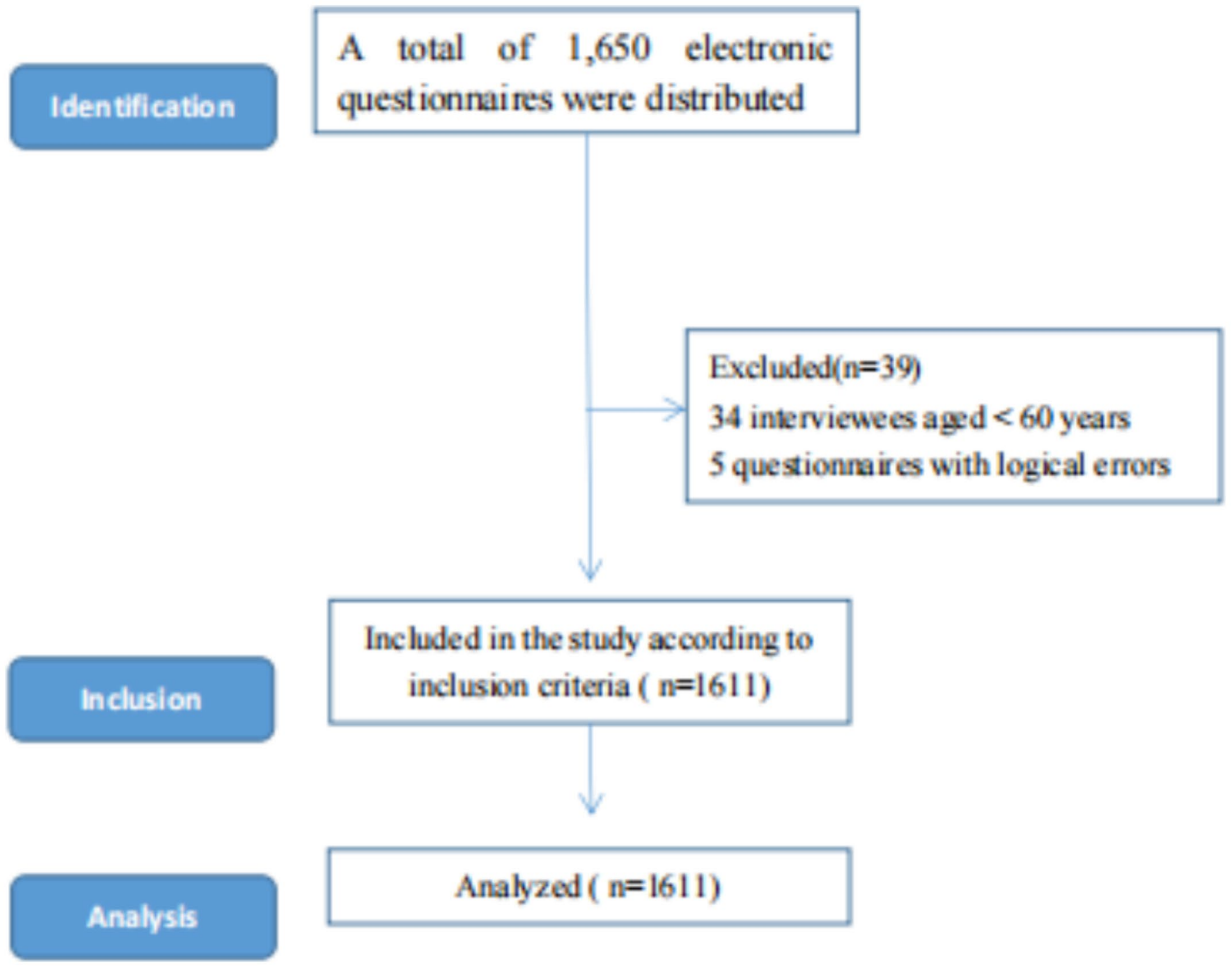
Sample selection process for this cross-sectional study.

Finally, 1,611 valid questionnaires were collected out of 1,650 distributed, resulting in a validity rate of 97.6%. The inclusion criteria were as follows: (1) registered residents of Anhui Province, (2) individuals aged ≥60 years, and (3) subjects who provided oral agreement and written informed consent. Participants were excluded if they had any of the following conditions: (1) dementia or cognitive impairment, (2) hearing or visual impairment, (3) serious mental and neurological diseases, or (4) were unwilling or unable to cooperate with the completion of the investigation owing to various uncontrollable reasons (Figure 1).

### Measures

#### Demographic information

A self-designed questionnaire was developed according to the objective of this study. The sociodemographic data (i.e., gender, age, occupation, marital status, educational level, household income, and residence status) of the enrolled participants were collected through a sociodemographic information sheet.

#### Frailty assessment

In this study, the FRAIL scale was used to assess the frailty of older adult patients with underlying chronic diseases. The FRAIL scale, a commonly used tool for screening frailty in older adults in clinical settings (17), was proposed by the expert group of the International Society for Geriatric Nutrition in 2001. Generally, this scale consists of five items: fatigue (experienced for most or all of the past 4 weeks); increased resistance/decreased endurance (difficulty climbing one flight of stairs without aid or assistance from another person); decline in free movement (difficulty walking 100 m without any auxiliary tools and without the help of others); underlying diseases [presence of at least five of the following diseases: hypertension, diabetes, coronary heart disease, stroke, malignant tumors (except for small skin tumors), congestive heart failure, asthma, arthritis, chronic lung disease, kidney disease, etc.]; and weight loss (weight loss of ≥5% within the past 1 year or less). Each “yes” response is scored as 1, and each “no” response is scored as 0. With a total score ranging from 0 to 5, individuals are classified as frail if their score is ≥3. The Chinese version of this scale has demonstrated high reliability and validity in older Chinese adults with chronic diseases (18). The Cronbach’s *α* was 0.86 in this study.

#### Sarcopenia

For the screening of sarcopenia, this study used the SARC-F (19, 20). This 5-item scale assesses subjects on aspects of strength (difficulty in handling a 10-pound load: 0 for no difficulty, 1 for occasional difficulties, and 2 for frequent difficulties or not at all), walking ability (same scoring for difficulty walking across the room), standing ability (same scoring for difficulty getting up from a bed or chair), stair climbing ability (same scoring for difficulty climbing 10 flights of stairs), and falls (number of falls in the past year: never = 0 points, 1–3 times = 1 point, and more than four times = 2 points). Each item is scored between 0 and 2, resulting in a total score from 0 to 10, with a total score of ≥4 indicating sarcopenia risk (15). The Cronbach’s *α* value of the SARC-F was 0.83 in this study.

#### Social frailty

The status of social frailty was evaluated using the social frailty scale. This scale consists of five items: inability to help others (I had not been able to help my friends or family in the previous 12 months), limited social participation (I had not participated in social or leisure time activities in the previous 12 months), loneliness (I have felt lonely in the past week), financial difficulty (I had not had enough income to live on for the previous 12 months.), and not having anyone to talk to. With a total score range of 0–5 points, social frailty can be identified in subjects with ≥3 points (21). The Cronbach’s α value of the social frailty scale was 0.85 in this study.

#### Malnutrition

The nutritional status of the enrolled participants was assessed by the short-form mini-nutritional assessment (MNA-SF), which has been validated for malnutrition screening in frail older adults (22). The MNA-SF includes six items: (1) weight loss in the past 3 months: >3 kg = 0, unknown = 1, 1–3 kg = 2, and no = 3; (2) body mass index: <19 = 0, 19–21 = 1, 21–23 = 2, and >23 = 3; (3) psychological stress or acute illness: no = 0 and yes = 2; (4) mobility: having been bedridden for a long time = 0, able but unwilling to be active = 1, and outdoor activities = 2; (5) neuropsychological problems: severe dementia or depression = 0, mild dementia or depression = 1, and no = 2; and (6) food intake: significantly reduced food intake = 0, slightly reduced food intake = 1, and no = 2. With a maximum total score of 14, this scoring would facilitate the identification of nutritional status, including good nutrition, risk of malnutrition, and malnutrition, with total scores of 12–14, 8–11, and 0–7 points, respectively. Cronbach’s α of the MNA-SF was 0.90 in this study.

#### Quality control

The investigation procedures and methods of this study were subjected to unified and strict control. The investigators were all medical students and underwent standardized training before participating in this survey. Paper questionnaires were used in this survey, which would contribute to careful double-checking during data entry and the exclusion of invalid questionnaires in a timely manner.

### Statistical analysis

This study used an electronic questionnaire system, i.e., Questionnaire Star, to collect the original data and used SPSS 22.0 software for data analysis after data entry. All descriptive data collected were summarized with numbers (%) for categorical variables and means±standard deviations (SDs) for continuous variables. The chi-squared (χ^2^) test was used to compare differences between sociodemographic characteristics and frailty. Pearson’s correlation analysis was conducted to investigate the associations between social frailty, sarcopenia, and malnutrition and frailty. Finally, a logistic regression model was used to analyze the influential factors of frailty (method: forward LR, entry: 0.05, removal: 0.10), with frailty status as the dependent variable (binary dependent variables: frail vs. non-frailty). R (version 4.3.1) was used to construct the model and evaluate the model quality. A nomogram predictive model was further established based on the significant influential factors, using the ‘rms’ package in R software. The performance of this nomogram was internally validated by bootstrap resampling (1,000 times) analysis. Additionally, the calibration and discrimination ability of the nomogram were evaluated using a calibration chart and the receiver operating characteristic (ROC) curve, and the corresponding clinical effectiveness was determined based on decision curve analysis (DCA). The final model was validated using an independent validation dataset. A two-sided *p*-value of <0.05 was considered to be statistically significant.

## Result

### Description of the sociodemographic characteristics of older adults

Table 1 presents the sociodemographic characteristics of the enrolled older adults. Among the 1,611 older adults, 57.7% (*n* = 930) were male and the rest were female, i.e., 42.3% (*n* = 681). The older adults ranged in age from 60 to 98 years old, with an average age of 70.82 ± 7.432.

**Table 1:** Univariate analysis of the participants’ demographics (*N* = 1,611).

Variables	Overall	Non-frailty	Frailty	*χ* ^2^	*p*
	1,611	*N* = 1,120 (69.5)	*N* = 491 (30.5)		
Sex				2.86	0.09
Male	930 (57.7)	662 (71.2)	268 (28.8)		
Female	681 (42.3)	458 (67.3)	223 (32.7)		
Age group				131.92	<0.001
60–69	755 (46.9)	620 (82.1)	135 (17.9)		
70–79	619 (38.4)	392 (63.3)	227 (36.7)		
≥80	237 (14.7)	108 (45.6)	129 (54.4)		
Hospitalization within 6 months				96.02	<0.001
No	532 (33.0)	455 (85.5)	77 (14.5)		
Yes	1,079 (67.0)	665 (61.6)	414 (38.4)		
Malnutrition				180.67	<0.001
No	1,048 (65.1)	847 (80.8)	201 (19.2)		
Yes	563 (34.9)	273 (48.5)	290 (51.5)		
Social frailty				281.69	<0.001
No	899 (55.8)	779 (86.7)	120 (13.3)		
Yes	712 (44.2)	341 (47.9)	371 (52.1)		
Sarcopenia				512.77	<0.001
No	1,142 (70.9)	984 (86.2)	158 (13.8)		
Yes	469 (29.1)	136 (29.0)	333 (71.0)		
Regular physical examination				1.32	0.25
No	598 (37.1)	426 (71.2)	172 (28.8)		
Yes	1,013 (62.9)	694 (68.5)	319 (31.5)		
Smoking				0.002	0.97
No	1,015 (63.0)	706 (69.6)	309 (30.4)		
Yes	596 (37.0)	414 (69.5)	182 (30.5)		
Drinking				2.08	0.15
No	1,011 (62.8)	690 (68.2)	321 (31.8)		
Yes	600 (37.2)	430 (71.7)	170 (28.3)		
Have contracted family doctor				0.52	0.47
No	76 (4.7)	50 (65.8)	26 (34.2)		
Yes	1,535 (95.3)	1,070 (69.7)	465 (30.3)		

### Univariate analysis of risk factors for frailty in older adults

In this study, 1,120 cases had no frailty, and 491 cases had frailty, with the prevalence of frailty being 30.5% (491/1,611) in older adults. There were significant differences in age group, hospitalization within 6 months, malnutrition, social frailty, and sarcopenia (all *p* < 0.001, Table 1).

### Binary analysis of the influential factors of frailty

To explore the influential factors of frailty, a binary logistic regression analysis was conducted, with age, hospitalization within 6 months, malnutrition, social frailty, and sarcopenia as independent variables and frailty (grouping, 1 = non-frailty and 2 = frailty) as the dependent variable. As shown in Table 2, frailty would occur in older adults with social frailty (OR = 2.74, 95% CI: 2.05–3.66), malnutrition (OR = 1.89, 95% CI: 1.42–2.51), sarcopenia (OR = 7.92, 95% CI: 5.96–10.54), hospitalization within 6 months (OR = 2.21, 95% CI: 1.58–3.09), and age ≥70 years old (OR = 1.72, 95% CI: 1.27–2.33; OR = 2.20, 95% CI: 1.48–3.27).

**Table 2:** Binary logistic regression analysis of influential factors associated with frailty.

Indices	*β*	Wald	*p*-value	OR	95% CI
Social frailty	1.01	0.15	< 0.001	2.74	2.05–3.66
Malnutrition	0.64	0.15	< 0.001	1.89	1.42–2.51
Sarcopenia	2.07	0.15	< 0.001	7.92	5.96–10.54
Hospitalization within 6 months	0.79	0.17	< 0.001	2.21	1.58–3.09
Age group			< 0.001		
70–79	0.54	0.16	< 0.001	1.72	1.28–2.33
≥80	0.79	0.20	< 0.001	2.20	1.48–3.27
Constant	−3.33	0.19	< 0.001	0.04	

### Predictive model development

**Figure 2:**
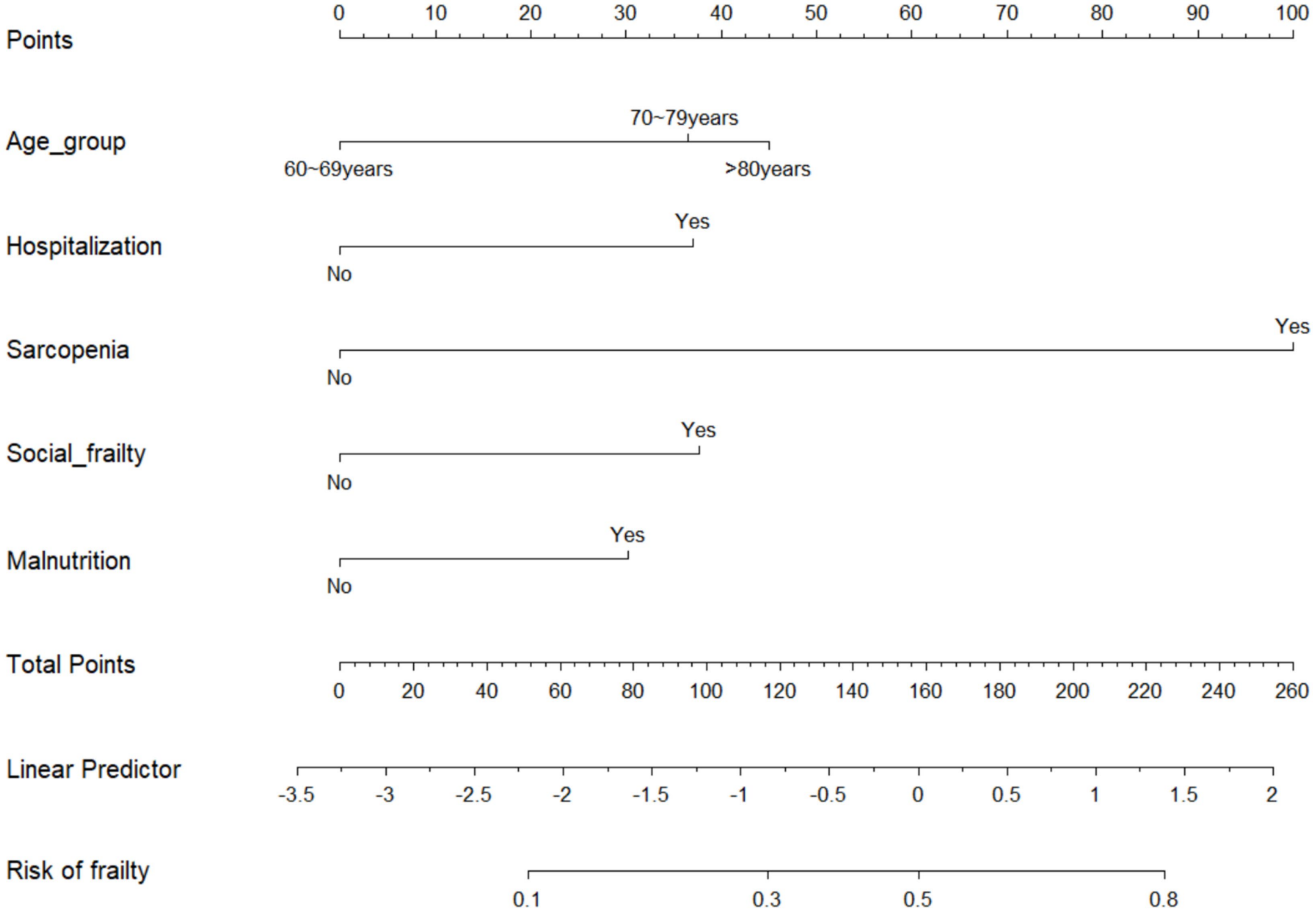
Nomogram model of frailty risk in older Chinese adults.

A one-way analysis of variance was performed to identify the optimal predictors of the model through multivariate analysis of the variables selected from univariate analysis. Meanwhile, the predictive model was constructed using a multivariate logistic regression analysis. The predictive model consisted of variables with statistically significant differences (*p* < 0.05) in the multivariate logistic regression. These variables included age group, sarcopenia, malnutrition, social frailty, and hospitalization as predictors. Finally, a predictive model was proposed using a nomogram, which may contribute to a quantitative prediction of the risk of frailty in older Chinese adults (Figure 2).

**Figure 3:**
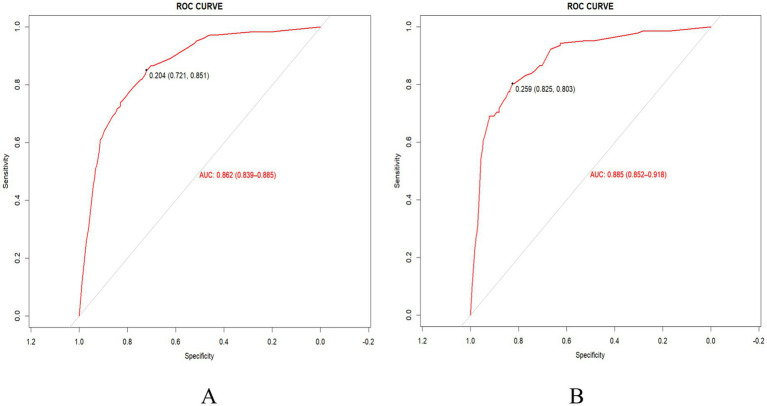
ROC curve and the AUC of the nomogram in the training set **(A)** and the validation set **(B)**.

By testing the incidence of frailty in older Chinese adults in the training set and the validation set, the area under the ROC curve (AUC) value was calculated to evaluate the discriminative performance of the predictive model. As shown in Figures 3A,B, the AUC values of the predictive model were 0.86 (95%CI 0.84–0.89) and 0.89 (95%CI 0.85–0.92) in the training and validation sets, respectively, with a specificity of 0.72 and 0.84, as well as a sensitivity of 0.85 and 0.80, respectively. Collectively, the nomogram established in our study exhibited good discriminative power and predictive value, which may facilitate the correct identification of frail and non-frail older Chinese adults.

### Calibration curve of the predictive model

**Figure 4:**
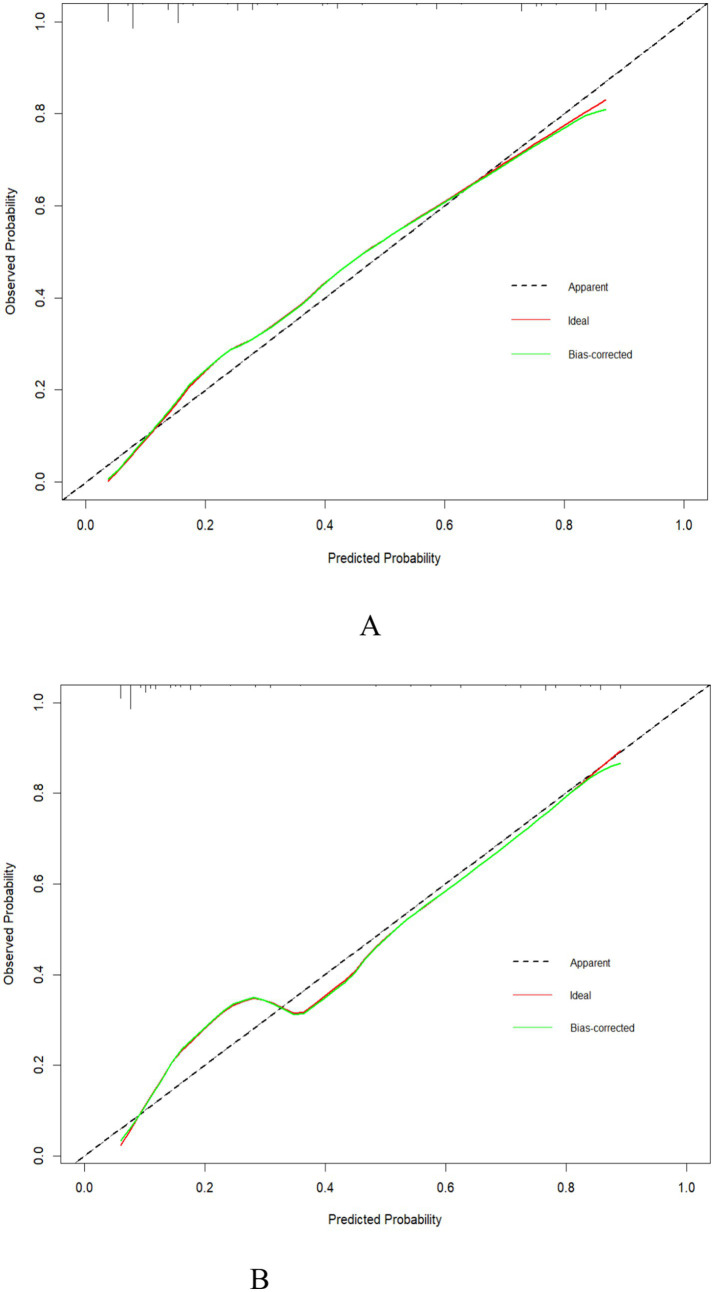
Calibration curve of the nomogram for the training set **(A)** and the validation set **(B)**.

In accordance with the results of the calibration plots (Figures 4A,B) and the Hosmer–Lemeshow goodness-of-fit test (*p* > 0.05 for very good fit), the model had a good fit for both the training set (*χ*2 = 12.02, df = 8, *p* = 0.15) and the validation set (*χ*2 = 11.67, df = 8, *p* = 0.167). Meanwhile, the calibration curves of the nomogram displayed a high degree of agreement between the predicted and actual probabilities of frailty in the training set (Figure 4A) and the validation set (Figure 4B).

### Evaluation of the clinical validity

**Figure 5:**
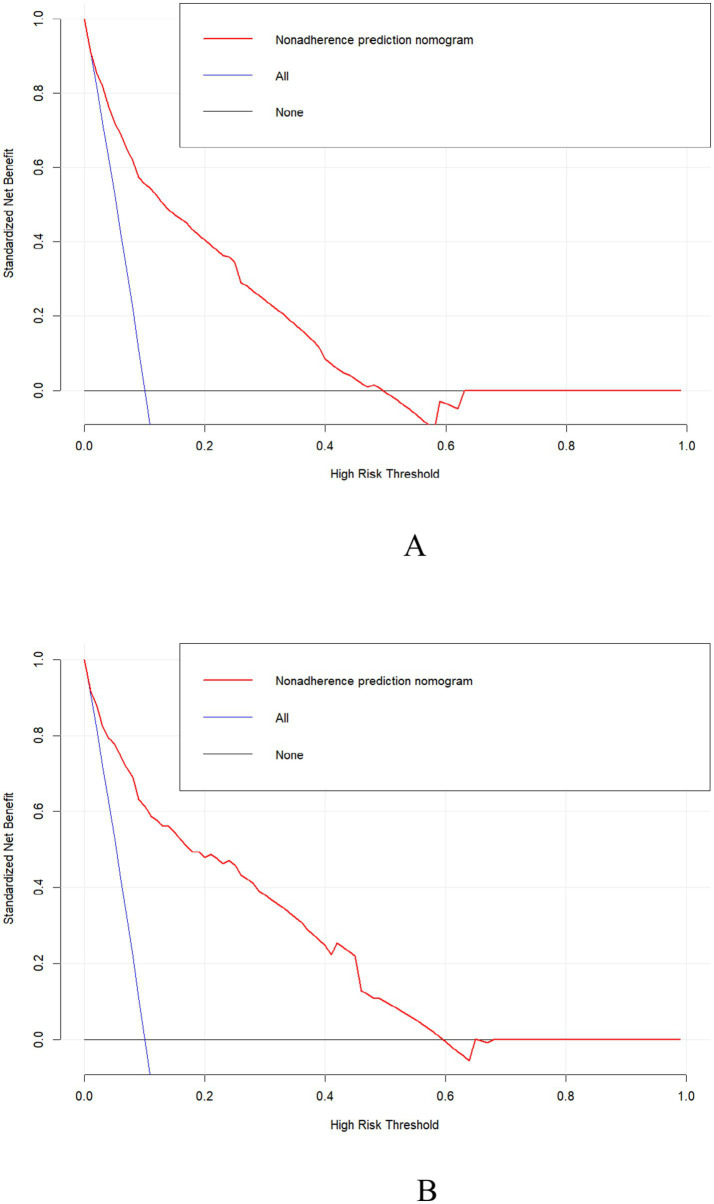
Clinical decision curves for the training set **(A)** and the validation set **(B)**.

DCA was used to evaluate the clinical validity of the model. As shown in Figures 5A,B, the net benefits of the predictive model for the internal validation set were significantly higher than those of the two extreme cases, indicating that the nomogram constructed in our study offers superior net benefit and predictive accuracy.

## Discussion

As a significant threat to China’s aging population, frailty has a serious impact on older adults’ quality of life and also places heavy economic pressure and caregiving burdens on families, affecting the harmonious and healthy development of society to a certain extent. Therefore, to inform public health policymakers and promote healthy aging among older adults, we conducted a cross-sectional study on frailty and risk factors among older adults in Anhui Province, China. In our study, the prevalence of frailty was 30.4% among older adults in Anhui Province, which is highly consistent with the rate reported by Liu et al. (23). Although this study used a convenience sampling method, the results indicate that the research subjects encompassed elderly individuals from urban, town, and rural areas in southern, central, and northern Anhui. Therefore, the impact of convenience sampling on study outcomes is minimal. In the future, we will adopt stratified sampling to enhance the generalizability of research. Siriwardhana et al. (24) found in their 2018 systematic review and meta-analysis that the prevalence of frailty among older adults ranged from 3.9 to 51.4% across different countries, which may be attributed to potential differences in the frailty assessment tools used and geographical regions. Fried et al. also proposed that frailty is an important risk factor for a range of adverse health outcomes and that identifying at-risk populations is critical for preventing frailty and reducing its associated adverse outcomes, especially in the early stage in older adults. In addition to being a manifestation of the decline in physiological function in older adults, frailty is also a key factor in predicting increased risks in the presence of stressful events. When confronted with stressors such as surgery, infections, and falls, frail older adults are prone to disability, prolonged hospitalization, and death, proposing higher requirements for long-term care and medical expenses (25). Therefore, it is of great significance to identify high-risk populations to prevent frailty and reduce related adverse outcomes (17).

Our research also revealed that frailty generally increased with age, which was consistent with the existing epidemiological survey (26). The results of this study align with those of a cross-sectional survey on frailty in community-dwelling older adults in northwest China.

With aging, degenerative changes in several physiological systems may induce decreased functioning and increased risk of frailty in older adults (27). The oldest-old may experience a high risk of frailty, necessitating dynamic frailty risk assessments and strengthened daily care for this group of participants. Additionally, the involvement of social, medical, and pension institutions should pay more attention to the oldest-old.

Furthermore, our study found that frail older adults had a higher risk of hospitalization than non-frail older adults, revealing a correlation between hospitalization and frailty in older adults, which was consistent with that of a longitudinal study (28). Frailty has been recognized as a key factor for hospitalization in older adults, who experience the highest risk of hospitalization in this group (29). Sharma Y. et al. observed a prevalence of frailty in a considerable proportion of hospitalized elderly patients (30). Frailty, such as malnutrition, may usually manifest as a decline in the reserve capacity of major organ systems, which is generally common among patients during hospitalization, one of the most stressful events for older adults (31). Older adults are primarily hospitalized due to gradually declining physical functions and a weakened immune system, rendering them more susceptible to infections and diseases. Such elevated vulnerability may elevate the risk of developing frailty. Therefore, longitudinal studies are required to explore the relationship between frailty in older adults and different hospitalization times.

In this study, sarcopenia was demonstrated to be associated with an increased risk of frailty in older Chinese adults, which was in accordance with that reported in a previous population-based cohort study (32). A growing body of evidence supports the relationship between sarcopenia and several adverse health outcomes, such as falls, physical frailty, and disability, in particular (33, 34). Older adults with sarcopenia may often experience progressive loss of muscle mass and poor muscle function, resulting in an increased likelihood of adverse health outcomes such as physical disability and compromised quality of life (32), which may explain the occurrence of frailty in this group to some extent. Meanwhile, sarcopenia and frailty may overlap in pathophysiological mechanisms (35). In a population-based cohort study, sarcopenia was observed to be a potential modulator of transitions in the status of frailty, as evidenced by the observation that sarcopenic individuals had more than (34). As a result, for older adults with sarcopenia, it may be beneficial to develop reasonable exercise and nutritional support strategies to effectively prevent the further development of sarcopenia (36). Evidence has underscored the significance of physical activity, particularly moderate-to-vigorous intensity physical activity, in significantly mitigating the progression of sarcopenia and frailty by enhancing muscle protein synthesis, improving insulin sensitivity, and reducing systemic inflammation (37, 38). Moreover, physical activity has been found to serve a dual role as both a preventive and therapeutic modality, offering a cost-effective means to mitigate the cascading adverse outcomes (i.e., from disability to mortality) of frailty and sarcopenia (39). Similarly, vitamin D supplementation (2,000 IU/day) would contribute to improved skeletal muscle index and grip strength in patients with decompensated cirrhosis, reducing the prevalence of sarcopenia from 80 to 33% over 12 months clinically (40). These findings suggest that early and effective intervention for sarcopenia can prevent further aggravation of frailty and adverse health outcomes.

Our study also observed an association between social frailty and frailty, as reported in a cross-sectional study from Japan (41). In a 4-year cohort study in non-frail community older adults, social vulnerability might contribute to frailty in a relatively short period of time (42). Meanwhile, social isolation was a risk factor for frailty progression in the English Longitudinal Study of Ageing (43). Social frailty can lead to adverse outcomes (e.g., disability and death) in older adults (12, 44). A study from China suggested a lower prevalence of social frailty in China, attributed possibly to traditional family-based support for older Chinese adults (45). In China, family members are the major caregivers for older adults in their daily lives. Therefore, in addition to giving more support and care, family members should also encourage and assist older adults to go out.

Similar to studies in Malaysia and Thailand (46, 47), this study also identified a strong association between malnutrition and frailty, a relationship that has been demonstrated in community-dwelling older adults (48, 49). Both undernutrition and obesity may increase the risk of frailty in community-dwelling older individuals (50, 51). Morley et al. proposed four main mechanisms that trigger frailty: atherosclerosis, sarcopenia, cognitive deterioration, and malnutrition, with their respective metabolic alterations (52). Mechanistically and critically, malnutrition can lead to weight loss and impaired physical functioning (53). Given its association with cognitive impairment or loss of function, malnutrition may cause cognitive frailty (52). Moreover, Kelaiditi et al. analyzed, by pooling relevant studies, that nutritional therapy can improve frailty and delay functional decline (54). Another study based on the incorporation of 1,345 French older adults confirmed that frailty was associated with protein intake of less than 1 g/kg/day (55). Notably, nutritional supplements can be effective in improving nutritional intake in malnourished older adults, including those with frailty (56). On this basis, it can be feasible to administer oral nutritional supplements and add high-protein foods to the daily diet of older adults with malnutrition, which may improve their nutritional status.

This study holds significant clinical implications for mitigating frailty among older adults in Anhui Province and similar settings. Our study develops a predictive model that may provide a practical tool for healthcare providers, particularly in primary care and geriatric settings, which may facilitate the proactive screening of older individuals at high risk of frailty during routine assessments. On a personal level, identifying high-risk individuals early may allow for timely, personalized interventions to improve patients’ physical function and strengthen mental health support for them. At a public health level, these findings highlight that priority should be given to modifiable risk factors in community-based elder care programs and health policies. While immediate application should focus on the situation in Anhui province, the methodology and core risk factors identified provide a valuable template for developing similar predictive tools in other regions of China, ultimately preventing frailty-associated adverse outcomes such as disability, hospitalization, and poor quality of life.

## Strengths and limitations

The present study has several limitations. First, this cross-sectional study could only establish a correlation. Hence, the results of this study should be validated by a further longitudinal study to systematically understand the process and laws of frailty in older adults. Second, potential recall bias might be introduced inevitably due to the dependence on self-reports from older adults when using several scales in this study. Third, this study used a convenience sampling method to recruit participants from certain communities in Anhui Province. While this approach is operationally convenient and efficient, its inherent nature implies that the sample may not fully represent the overall characteristics of all elderly individuals in Anhui Province. This could result in an insufficient representation of certain specific subgroups (such as those residing in remote rural areas, those with poorer health status, those with limited mobility, or those with lower willingness to participate) in the study findings. Consequently, the generalizability of the frailty-related factors identified in this study and its predictive model may be limited; therefore, extra caution is warranted when extrapolating the results to a broader elderly population in Anhui Province and even other regions across the country. However, and importantly, this study is the first to investigate the relationship between frailty and social frailty based on the HALFT scale in China. Findings in this study may provide valuable reference for improving frailty in older adults and for effectively preventing and delaying the occurrence and deterioration of frailty.

## Conclusion

To sum up, this study reveals a relatively high prevalence of frailty among older adults in Anhui, China. The predominant risk factors for frailty in this population may include hospitalization within the past 6 months, malnutrition, sarcopenia, and social frailty. Given its negative impact on increasing the risk of adverse events among older adults, frailty should be managed or prevented through the implementation of effective community-, hospital-, and home-based early assessments and interventions, thereby reducing the risk of frailty in older adults and delaying the progression of this disease.

## Data Availability

The raw data supporting the conclusions of this article will be made available by the authors, without undue reservation.

## References

[1] Li G Wang C Guan X Bai Y Feng Y Wei W. Age-related DNA methylation on Y chromosome and their associations with total mortality among Chinese males. Aging Cell. (2022) 21:e13563. doi: 10.1111/acel.13563, PMID: 35120273 PMC8920452

[2] Dent E Martin FC Bergman H Woo J Romero-Ortuno R Walston JD. Management of frailty: opportunities, challenges, and future directions. Lancet. (2019) 394:1376–86. doi: 10.1016/S0140-6736(19)31785-4, PMID: 31609229

[3] Kojima G Liljas AEM Iliffe S. Frailty syndrome: implications and challenges for health care policy. Risk Manag Healthc Policy. (2019) 12:23–30. doi: 10.2147/RMHP.S168750, PMID: 30858741 PMC6385767

[4] Walsh B Fogg C Harris S Roderick P de Lusignan S England T. Frailty transitions and prevalence in an ageing population: longitudinal analysis of primary care data from an open cohort of adults aged 50 and over in England, 2006-2017. Age Ageing. (2023) 52:afad058. doi: 10.1093/ageing/afad058, PMID: 37140052 PMC10158172

[5] Hoogendijk EO Afilalo J Ensrud KE Kowal P Onder G Fried LP. Frailty: implications for clinical practice and public health. Lancet. (2019) 394:1365–75. doi: 10.1016/S0140-6736(19)31786-6, PMID: 31609228

[6] Travers J Romero-Ortuno R Langan J MacNamara F McCormack D McDermott C. Building resilience and reversing frailty: a randomised controlled trial of a primary care intervention for older adults. Age Ageing. (2023) 52:d12. doi: 10.1093/ageing/afad012 36849160

[7] Boucham M Salhi A El Hajji N Gbenonsi GY Belyamani L Khalis M. Factors associated with frailty in older people: an umbrella review. BMC Geriatr. (2024) 24:737. doi: 10.1186/s12877-024-05288-4, PMID: 39237866 PMC11376099

[8] Leij-Halfwerk S Verwijs MH van Houdt S Borkent JW Guaitoli PR Pelgrim T. Prevalence of protein-energy malnutrition risk in European older adults in community, residential and hospital settings, according to 22 malnutrition screening tools validated for use in adults ≥65 years: a systematic review and meta-analysis. Maturitas. (2019) 126:80–9. doi: 10.1016/j.maturitas.2019.05.006, PMID: 31239123

[9] Celik HI Koc F Siyasal K Ay B Ilter NB Celik OM. Exploring the complex associations among risks of malnutrition, sarcopenia, and frailty in community-dwelling older adults. Eur Rev Aging Phys Act. (2024) 21:18. doi: 10.1186/s11556-024-00354-7, PMID: 38982337 PMC11232342

[10] Wang C Guo X Xu X Liang S Wang W Zhu F. Association between sarcopenia and frailty in elderly patients with chronic kidney disease. J Cachexia Sarcopenia Muscle. (2023) 14:1855–64. doi: 10.1002/jcsm.13275, PMID: 37300354 PMC10401549

[11] Zare H Tagharrobi Z Zare M. Cross-cultural adaptation and psychometric evaluation of the social frailty scale in Iranian older adults. BMC Geriatr. (2024) 24:368. doi: 10.1186/s12877-024-04940-3, PMID: 38658817 PMC11040830

[12] Makizako H Shimada H Tsutsumimoto K Lee S Doi T Nakakubo S. Social frailty in community-dwelling older adults as a risk factor for disability. J Am Med Dir Assoc. (2015) 16:1003.e7–1003.e12. doi: 10.1016/j.jamda.2015.08.023 26482055

[13] Ragusa FS Veronese N Smith L Koyanagi A Dominguez LJ Barbagallo M. Social frailty increases the risk of all-cause mortality: a longitudinal analysis of the English longitudinal study of ageing J. Exp Gerontol. (2022) 167:111901. doi: 10.1016/j.exger.2022.111901, PMID: 35870753

[14] Zhang H Hu Z Jiang S Hao M Li Y Liu Y. Social frailty and the incidence of motoric cognitive risk syndrome in older adults. Alzheimers Dement. (2024) 20:2329–39. doi: 10.1002/alz.13696, PMID: 38284799 PMC11032557

[15] Choi K Ko Y. The relationship between social frailty and cognitive impairment among older adults: the role of various types of internet use. Front Public Health. (2024) 12:1424465. doi: 10.3389/fpubh.2024.1424465, PMID: 39310909 PMC11412848

[16] Liu B Zhang X Jia S Wang W Huang J Kang L. Association of foods consumption and physical activity with prefrailty and frailty among Chinese older adults in urban communities: a cross-sectional study. Asia Pac J Clin Nutr. (2024) 33:447–56. doi: 10.6133/apjcn.202409_33(3).0015, PMID: 38965732 PMC11397568

[17] Fried LP Tangen CM Walston J Newman AB Hirsch C Gottdiener J. Frailty in older adults: evidence for a phenotype. J Gerontol A Biol Sci Med Sci. (2001) 56:M146–56. doi: 10.1093/gerona/56.3.m146, PMID: 11253156

[18] Ye B Gao J Fu H. Associations between lifestyle, physical and social environments and frailty among Chinese older people: a multilevel analysis. BMC Geriatr. (2018) 18:314. doi: 10.1186/s12877-018-0982-1, PMID: 30547760 PMC6295038

[19] Barbosa-Silva TG Menezes AM Bielemann RM Malmstrom TK Gonzalez MC. Enhancing SARC-F: improving sarcopenia screening in the clinical practice. J Am Med Dir Assoc. (2016) 17:1136–41. doi: 10.1016/j.jamda.2016.08.004, PMID: 27650212

[20] Yao R Yao L Yuan C Gao BL. Accuracy of calf circumference measurement, SARC-F questionnaire, and Ishii's score for screening stroke-related sarcopenia. Front Neurol. (2022) 13:880907. doi: 10.3389/fneur.2022.880907, PMID: 35572926 PMC9099210

[21] Noguchi T Nojima I Inoue-Hirakawa T Sugiura H. Association between social frailty and sleep quality among community-dwelling older adults: a cross-sectional study. Phys Ther Res. (2021) 24:153–62. doi: 10.1298/ptr.E10085, PMID: 34532211 PMC8419475

[22] Hong X Yan J Xu L Shen S Zeng X Chen L. Relationship between nutritional status and frailty in hospitalized older patients. Clin Interv Aging. (2019) 14:105–11. doi: 10.2147/CIA.S189040, PMID: 30666096 PMC6330965

[23] Liu H Li YZ Zhang H Xiong M Zhang YP. The relationship between frailty and cognitive functioning in community-dwelling older adults: a mediating mechanism of psychological resilience. Pract Geriatr. (2022) 36:842–5.

[24] Siriwardhana DD Hardoon S Rait G Weerasinghe MC Walters KR. Prevalence of frailty and prefrailty among community-dwelling older adults in low-income and middle-income countries: a systematic review and meta-analysis. BMJ Open. (2018) 8:e018195. doi: 10.1136/bmjopen-2017-018195, PMID: 29496895 PMC5855322

[25] Cesari M Prince M Thiyagarajan JA de Carvalho IA Bernabei R Chan P. Frailty: an emerging public health priority. J Am Med Dir Assoc. (2016) 17:188–92. doi: 10.1016/j.jamda.2015.12.016, PMID: 26805753

[26] Yu X Shi Z Wang D Niu Y Xu C Ma Y. Prevalence and associated factors of frailty among community dwelling older adults in Northwest China: a cross-sectional study. BMJ Open. (2022) 12:e060089. doi: 10.1136/bmjopen-2021-060089, PMID: 35914908 PMC9345078

[27] Rockwood K Howlett SE. Age-related deficit accumulation and the diseases of ageing. Mech Ageing Dev. (2019) 180:107–16. doi: 10.1016/j.mad.2019.04.005, PMID: 31002924

[28] Theou O Sluggett JK Bell JS Lalic S Cooper T Robson L. Frailty, hospitalization, and mortality in residential aged care. J Gerontol A Biol Sci Med Sci. (2018) 73:1090–6. doi: 10.1093/gerona/glx185, PMID: 29985993

[29] Chang SF Lin HC Cheng CL. The relationship of frailty and hospitalization among older people: evidence from a meta-analysis. J Nurs Scholarsh. (2018) 50:383–91. doi: 10.1111/jnu.12397, PMID: 29874399

[30] Sharma Y Avina P Ross E Horwood C Hakendorf P Thompson C. The overlap of frailty and malnutrition in older hospitalised patients: an observational study. Asia Pac J Clin Nutr. (2021) 30:457–63. doi: 10.6133/apjcn.202109_30(3).0012, PMID: 34587705

[31] Tseng HK Cheng YJ Yu HK Chou KT Pang CY Hu GC. Malnutrition and frailty are associated with a higher risk of prolonged hospitalization and mortality in hospitalized older adults. Nutrients. (2025) 17:221. doi: 10.3390/nu17020221, PMID: 39861351 PMC11767747

[32] Davies B García F Ara I Artalejo FR Rodriguez-Mañas L Walter S. Relationship between sarcopenia and frailty in the Toledo study of healthy aging: a population based cross-sectional study. J Am Med Dir Assoc. (2018) 19:282–6. doi: 10.1016/j.jamda.2017.09.014, PMID: 29079029

[33] Marzetti E Calvani R Tosato M Cesari M Di Bari M Cherubini A. Sarcopenia: an overview. Aging Clin Exp Res. (2017) 29:11–7. doi: 10.1007/s40520-016-0704-5, PMID: 28155183

[34] Álvarez-Bustos A Carnicero-Carreño JA Davies B Garcia-Garcia FJ Rodríguez-Artalejo F Rodríguez-Mañas L. Role of sarcopenia in the frailty transitions in older adults: a population-based cohort study. J Cachexia Sarcopenia Muscle. (2022) 13:2352–60. doi: 10.1002/jcsm.13055, PMID: 35903871 PMC9530539

[35] Perazza LR Brown-Borg HM Thompson LV. Physiological systems in promoting frailty. Compr Physiol. (2022) 12:3575–620. doi: 10.1002/cphy.c210034, PMID: 35578945 PMC9531553

[36] Chang KV Wu WT Huang KC Han DS. Effectiveness of early versus delayed exercise and nutritional intervention on segmental body composition of sarcopenic elders - a randomized controlled trial. Clin Nutr. (2021) 40:1052–9. doi: 10.1016/j.clnu.2020.06.037, PMID: 32723507

[37] Dodds R Sayer AA. Sarcopenia and frailty: new challenges for clinical practice. Clin Med (Lond). (2016) 16:455–8. doi: 10.7861/clinmedicine.16-5-455, PMID: 27697810 PMC6297299

[38] Liang C Shi L Li B He Z. The mediating role of sarcopenia in the association between physical activity and falls among Chinese older adults: a cross-sectional study. Healthcare (Basel). (2023) 11. doi: 10.3390/healthcare11243146, PMID: 38132036 PMC10743279

[39] Shuval K Leonard T Drope J Katz DL Patel AV Maitin-Shepard M. Physical activity counseling in primary care: insights from public health and behavioral economics. CA Cancer J Clin. (2017) 67:233–44. doi: 10.3322/caac.21394, PMID: 28198998

[40] Okubo T Atsukawa M Tsubota A Ono H Kawano T Yoshida Y. Effect of vitamin D supplementation on skeletal muscle volume and strength in patients with decompensated liver cirrhosis undergoing branched chain amino acids supplementation: a prospective, randomized, controlled pilot trial. Nutrients. (2021) 13:1874. doi: 10.3390/nu13061874, PMID: 34070910 PMC8228227

[41] Hironaka S Kugimiya Y Watanabe Y Motokawa K Hirano H Kawai H. Association between oral, social, and physical frailty in community-dwelling older adults. Arch Gerontol Geriatr. (2020) 89:104105. doi: 10.1016/j.archger.2020.104105, PMID: 32480111

[42] Makizako H Shimada H Doi T Tsutsumimoto K Hotta R Nakakubo S. Social frailty leads to the development of physical frailty among physically non-frail adults: a four-year follow-up longitudinal cohort study. Int J Environ Res Public Health. (2018) 15:490. doi: 10.3390/ijerph15030490, PMID: 29534470 PMC5877035

[43] Gale CR Westbury L Cooper C. Social isolation and loneliness as risk factors for the progression of frailty: the English longitudinal study of ageing. Age Ageing. (2018) 47:392–7. doi: 10.1093/ageing/afx188, PMID: 29309502 PMC5920346

[44] Yamada M Arai H. Social frailty predicts incident disability and mortality among community-dwelling Japanese older adults. J Am Med Dir Assoc. (2018) 19:1099–103. doi: 10.1016/j.jamda.2018.09.013, PMID: 30471801

[45] MaLSunFTangZ. Social frailty is associated with physical functioning, cognition, and depression, and predicts mortality. J Nutr Health Aging. (2018) 22:989–95. doi: 10.1007/s12603-018-1054-0, PMID: 30272104

[46] Norazman CW Adznam SN Jamaluddin R. Malnutrition as key predictor of physical frailty among Malaysian older adults. Nutrients. (2020) 12:1713. doi: 10.3390/nu12061713, PMID: 32521618 PMC7352933

[47] Seesen M Sirikul W Ruangsuriya J Griffiths J Siviroj P. Cognitive frailty in Thai community-dwelling elderly: prevalence and its association with malnutrition. Nutrients. (2021) 13:4239. doi: 10.3390/nu13124239, PMID: 34959791 PMC8709040

[48] Luger E Haider S Kapan A Schindler K Lackinger C Dorner TE. Association between nutritional status and quality of life in (pre) frail community-dwelling older persons. J Frailty Aging. (2016) 5:141–8. 29239581

[49] Chye L Wei K Nyunt MSZ Gao Q Wee SL Ng TP. Strong relationship between malnutrition and cognitive frailty in the Singapore longitudinal ageing studies (SLAS-1 and SLAS-2). J Prev Alzheimers Dis. (2018) 5:142–8. doi: 10.14283/jpad.2017.46, PMID: 29616708 PMC12280758

[50] Hubbard RE Lang IA Llewellyn DJ Rockwood K. Frailty, body mass index, and abdominal obesity in older people. J Gerontol A Biol Sci Med Sci. (2010) 65:377–81. doi: 10.1093/gerona/glp186, PMID: 19942592

[51] BollweinJVolkertDDiekmannRKaiserMJUterWVidalK. Nutritional status according to the mini nutritional assessment (MNA®) and frailty in community dwelling older persons: a close relationship. J Nutr Health Aging. (2013) 17:351–6. doi: 10.1007/s12603-013-0034-7, PMID: 23538658

[52] Gómez-Gómez ME Zapico SC. Frailty, cognitive decline, neurodegenerative diseases and nutrition interventions. Int J Mol Sci. (2019) 20:2842. doi: 10.3390/ijms20112842, PMID: 31212645 PMC6600148

[53] Verlaan S Ligthart-Melis GC Wijers SLJ Cederholm T Maier AB de van der Schueren MAE. High prevalence of physical frailty among community-dwelling malnourished older adults-a systematic review and Meta-analysis. J Am Med Dir Assoc. (2017) 18:374–82. doi: 10.1016/j.jamda.2016.12.074, PMID: 28238676

[54] Kelaiditi E Van Kan GA Cesari M. Frailty: role of nutrition and exercise. Curr Opin Clin Nutr Metab Care. (2014) 17:32–9. doi: 10.1097/MCO.0000000000000008, PMID: 24281373

[55] Rahi B Colombet Z Gonzalez-Colaço Harmand M Dartigues JF Boirie Y Letenneur L. Higher protein but not energy intake is associated with a lower prevalence of frailty among community-dwelling older adults in the French Three-City cohort. J Am Med Dir Assoc. (2016) 17:672.e7–672.e11. doi: 10.1016/j.jamda.2016.05.005, PMID: 27346652

[56] Nieuwenhuizen WF Weenen H Rigby P Hetherington MM. Older adults and patients in need of nutritional support: review of current treatment options and factors influencing nutritional intake. Clin Nutr. (2010) 29:160–9. doi: 10.1016/j.clnu.2009.09.003, PMID: 19828215

